# Diagnostic Approach to Ocular Toxoplasmosis

**DOI:** 10.3109/09273948.2011.595872

**Published:** 2011-07-19

**Authors:** Justus G Garweg, Jolanda DF de Groot-Mijnes, Jose G Montoya

**Affiliations:** 1 Swiss Eye Institute and University of Bern, Bern, Switzerland; 2 Department of Virology and Department of Ophthalmology, University Medical Center, Utrecht, The Netherlands; 3 Palo Alto Medical Foundation Toxoplasma Serology Laboratory, Palo Alto, California, USA and Department of Medicine and Division of Infectious Diseases and Geographic Medicine, Stanford University School of Medicine, Stanford, California, USA

**Keywords:** aqueous humor, autoimmunity, blood–aqueous barrier, ELISA, humoral immune response, immunoblotting, laboratory confirmation, local antibody production, ocular toxoplasmosis, PCR, retinochoroiditis, *Toxoplasma gondii*, uveovascular barrier, vitreous fluid

## Abstract

Toxoplasmic retinochoroiditis is deemed a local event, which may fail to evoke a detectable systemic immune response. A correct diagnosis of the disease is a necessary basis for estimating its clinical burden. This is not so difficult in a typical clinical picture. In atypical cases, further diagnostic efforts are to be installed. Although the aqueous humor may be analyzed for specific antibodies or the presence of parasitic DNA, the DNA burden therein is low, and in rare instances a confirmation would necessitate vitreous sampling. A laboratory confirmation of the diagnosis is frustrated by individual differences in the time elapsing between clinical symptoms and activation of specific antibody production, which may result in false negatives. In congenital ocular toxoplasmosis, a delay in the onset of specific local antibody production could reflect immune tolerance. Herein, the authors attempt to provide a simple and practicable algorithm for a clinically tailored diagnostic approach in atypical instances.

Toxoplasmosis is one of the commonest global zoonoses. In adults, the seroprevalence of antibodies against *Toxoplasma gondii* range in an age-dependent manner from 22.5% to more than 80% [1–5]. Our poor understanding of the pathophysiology of ocular toxoplasmosis is mirrored by our inability to unequivocally confirm a clinical diagnosis on the basis of laboratory tests. Although the clinical manifestations of the disease are usually highly characteristic, atypical manifestations are not uncommon, and these are not always recognized as specific of ocular toxoplasmosis even by experienced ophthalmologists. This circumstance raises questions as to the sensitivity and specificity of the clinical diagnosis, which, in the absence of a sufficiently sensitive laboratory test for the disease, is still regarded as the gold standard [6].

Although the diagnosis of ocular toxoplasmosis can be aided by the results of serological tests, these are not in themselves conclusive. Patients with ocular toxoplasmosis always register positive for *Toxoplasma*-specific IgG; but so, too, do infected individuals who manifest no signs of ocular involvement. Hence, the detection of *Toxoplasma*-specific IgG is of low diagnostic value [7, 8]. In some patients, *Toxoplasma*-specific IgM can be detected in the serum, which may be indicative of a recently acquired infection. However, in cases of acute infection equivocal or positive results are in itself not of diagnostic value. If the serological data confirm the existence of a recently acquired infection, then the alternative of a reactivated latent condition can be excluded. The absence of specific antibodies affords strong evidence against a toxoplasmic origin of the ocular disease. The parasite itself has been detected in the peripheral blood both of patients with ocular toxoplasmosis and of control individuals [9]. Therefore, the presence of specific antibodies or of the parasite in peripheral blood is not confirmative of ocular involvement.

## INTRAOCULAR PRESENCE OF THE PARASITE

Specific DNA can be detected in the intraocular fluids of patients with ocular toxoplasmosis using the polymerase chain reaction (PCR) technique. Although this method is usually a highly sensitive means of detecting nucleic acids, no standardized tests are available for the evaluation of ocular toxoplasmosis. Consequently, it is well-nigh impossible to assess the sensitivity and specificity of published data [10]. In immunocompetent individuals, *Toxoplasma* DNA can be amplified within samples of aqueous humor in maximally 30–40% of the clinically diagnosed cases [11–17]. In immunocompromised individuals, on the other hand, *Toxoplasma* DNA can be amplified in 75% of the clinically diagnosed cases [12, 13, 18]. The poor confirmation rate in immunocompetent patients suggests that, at the time when the clinical symptoms first become manifest, it is not the activity of the parasite itself but rather the host's immune response that drives the inflammatory process. The low DNA-amplification rates could also reflect a low parasitic burden in the aqueous humor (even in cases of acute infection), the smallness of the samples that are available for analysis, and/or an early degradation of *Toxoplasma* DNA [19, 20].

As an alternative to aqueous humor, aliquots of the vitreous can be analyzed. In samples of this liquor, parasitic DNA has been amplified in up to 50% of immunocompetent patients with clinically diagnosed ocular toxoplasmosis [21]. However, the withdrawal of samples of this ocular medium is justified only in severe atypical or complicated cases and in patients who are irresponsive to anti-*Toxoplasma* treatment. Notwithstanding, even the PCR technique is insufficiently sensitive to justify its choice as the sole laboratory test. In doubtful cases, it is advisable to analyze both the aqueous humor and the vitreous for the presence of parasitic DNA and of *Toxoplasma*-specific antibodies.

## LOCAL IMMUNE RESPONSE AND PRODUCTION OF SPECIFIC ANTIBODIES

In the context of active infection, a breakdown of the blood–retinal barrier may thwart a confirmation of the intraocular production of specific antibodies [22–25], since antibodies of the IgM type are rarely detected [26]. *Toxoplasma* tachyzoites are presumed to lodge within the retina during the primary infectious parasitemia. In most instances, they precipitate an ocular affection only during their reactivation within the retinal tissue. However, in certain global regions, ocular involvement occurs in a high proportion of cases during the initial invasion of the retina with the parasite [27, 28].

The detection of specific antibodies in intraocular fluids by the enzyme-linked immunosorbent assay (ELISA) technique is still deemed to be the gold standard for a laboratory confirmation of clinically diagnosed cases of ocular toxoplasmosis [20, 25, 29–34]. A common method to estimate the local versus systemic *Toxoplasma*-specific IgG is the Goldmann-Witmer coefficient. This index expresses the level of *Toxoplasma*-specific IgG relative to the level of total IgG in the aqueous humor as a fraction of the level of *Toxoplasma*-specific IgG relative to the level of the total IgG in the serum. A value of 2 or above is generally taken as evidence of the intraocular synthesis of *Toxoplasma*-specific IgG in response to the presence of the at a local site replicating tachyzoite. The sensitivity and the specificity of intraocular antibody detection have been reported to be 63 and 89%, respectively [35], although positivity rates up to 95% have been reported as well [17, 20, 32, 36]. One recognized practical problem of ocular fluid analysis lies in the inherently small volume of the samples that can be withdrawn [19], and this drawback is exacerbated by the low antibody levels that are usually present within these media. When the blood–retinal barrier is violated, the intraocular fluids are swamped with serum antibodies, high levels of which may mask the more subtle production in the ocular compartments [32].

The level of antibody production within the aqueous humor is governed by several unknown factors and can vary greatly between individuals suffering from ocular toxoplasmosis [15, 36]. In patients who have contracted the disease congenitally, the levels of *Toxoplasma*-specific antibodies within the serum do not increase with time after the onset of the symptoms, whereas antibodies within the aqueous humor do. In individuals with a recently acquired form of the disease, both the serum and the aqueous humor levels of anti-*Toxoplasma* antibodies are elevated.

## IMMUNOBLOTTING IN THE LABORATORY DIAGNOSIS OF OCULAR TOXOPLASMOSIS

**FIGURE 1:**
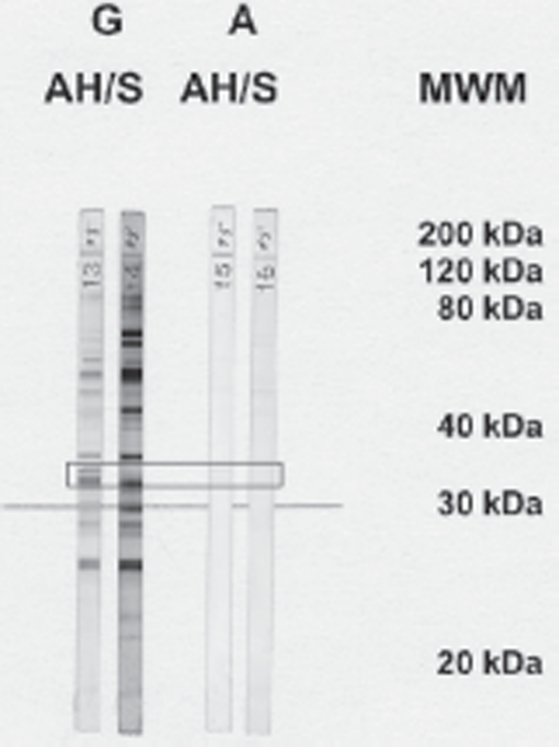
Recognition of toxoplasmal antigens by specific antibodies of the IgG (G) and IgA (A) type in samples of aqueous humor (AH) and serum (S) that were derived from a patient with acute ocular toxoplasmosis. The boxed region corresponds to an antigen size of 30 kDa, which is the most relevant one in the context of infection with *Toxoplasma*. Bands that are detected by immunoblotting in the aqueous humor but not in the serum correspond to antibodies that are produced locally but not systemi-cally. MWM, molecullar weight marker (in kDa).

Given the unsatisfactorily low sensitivity of available tests for the intraocular detection of antibody production in cases of ocular toxoplasmosis, the potential of immunoblotting has been pursued as an alternative technique (Figure 1) [37–40]. With this tool, local antibody production is presumed to have occurred if particular blot-bands are detected in the aqueous humor but not in the serum. The bands usually correspond to antibodies of the IgG type, although the IgM or IgA classes are also represented. In our hands, antibodies of the IgM class are disclosed in only 2% of cases in the absence of bands for IgG. Hence, immunoblotting for IgM is not sufficiently specific to be useful. Immunoblotting for IgA alone confirmed the clinical diagnosis in 23% of cases, and when this was combined with that for IgG, the percentage rose to 65% [40]. The failure to detect local antibody production in the remaining 35% of cases raises a question as to whether the inflammatory activity was systemic rather than local in these instances. Evidence in support of this contention is provided by an observation that PCR amplification of *Toxoplasma*-specific DNA yields a higher percentage of positive results in samples of blood than of aqueous humor [9]. However, the immune status of the patient may also influence the results. Indeed, samples of aqueous humor have been shown to register positive for *Toxoplasma* DNA more frequently in immunosuppressed than in immunologically healthy individuals [13].

## RELEVANCE OF AN INTACT BLOOD-RETINAL BARRIER

A comparison of the levels of anti-*Toxoplasma* antibodies within the serum and aqueous humor of Swiss patients—who were presumed to have been infected congenitally—with those of individuals living in the southern Brazilian region of Erechim—who were presumed to have acquired the disease postnatally—revealed higher levels in the latter than in the former group. Although the concentration of total (viz., *Toxoplasma*-specific and unspecific) IgG in the serum did not differ significantly between the two groups, the level of total IgG in the aqueous humor was markedly higher in the Brazilian than in the Swiss patients [41]. This phenomenon may reflect the degree of breakdown of the blood–retinal barrier [42, 43]. Accordingly, in patients with postnatally acquired ocular toxoplasmosis, a significant spillover of antibodies from the serum must be anticipated, and this event may partially account for the false-negative results that are yielded by tests for local antibody production [19, 32]. False-negative results can also arise if the infection is insufficiently strong to induce local antibody production, for instance, if the patient is immunocompromized or if the individual has developed an immune tolerance to the parasite, which is assumed for the congenitally contracted form of the disease [13, 44–46]. In the face of clinical evidence for disease activity, false-negative Goldmann-Witmer coefficients are obtained in more than 30% of cases. These may be accounted for either by a rapid breakdown of the blood–retinal barrier or by an early and strong systemic immune response to the parasite, which directs the clinical course of the ocular disease.

It must be borne in mind that the immune control of the parasite is mediated by the Thl-lymphocyte and not by a humoral response. This circumstance accounts for the increased risk of reactivation in patients who have undergone immunosuppressive therapy and in individuals who are suffering from a cellular immune deficiency. Against this background, it is somewhat surprising that our understanding of the cellular control of primary *Toxoplasma* infection and its reactivation is so much poorer than that of the host's humoral immune response to the parasite. In a clinical setting, the analysis of cytokine- and cell-mediated immune responses has yielded no information that could be usefully applied to routine practice [47–52].

## TIME LAPSE BETWEEN THE ONSET OF SYMPTOMS AND THE INDUCTION OF A LOCAL IMMUNE RESPONSE

In a significant proportion of patients (21%), a clinically relevant time lapse has to be expected between the onset of clinical symptoms and the activation of local antibody production, and this circumstance has been confirmed in a lapine model of ocular toxoplasmosis [53). In infection-naïve animals, the time lapse between inoculation with tachyzoites of *Toxoplasma gondii* and the local activation of specific antibody production may be up to 2 weeks. And even after marked infiltration of the vitreous, the interval is still 10 days. However, in a lapine model of secondary ocular toxoplasmosis, a delay of up to 3 weeks after the onset of symptoms can be expected. On the other hand, intraocular production of antibodies has been detected within the first 2 weeks of disease activity both in patients with primary ocular toxoplasmosis and in those with a recurrent manifestation of the disease. These findings indicate that the Goldmann-Witmer coefficient may still be a useful parameter in the early diagnosis of ocular toxoplasmosis [20].

## LIMITATIONS IN OUR UNDERSTANDING OF THE LOCAL IMMUNE RESPONSE

Several aspects relating to the laboratory diagnosis of ocular toxoplasmosis are poorly understood. Local antibody production cannot be confirmed in one-third of clinically diagnosed cases. This circumstance may reflect individual differences in the diagnostic window between the onset of symptoms and an activation of the local humoral immune response, the immune status of the patient, or a congenital immunotolerance, for which there is some theoretical support. Discrepancies also exist in the confirmation of local antibody production according to the ELISA and the immunoblotting techniques. Theoretically, the results of the two analyses should completely coincide. Consequently, the discrepancies are hardly explainable if not by infection with another agent, which has always to be considered in the differential diagnosis. *Toxocara* has been described to cause *Toxoplasma*-like lesions [54]. And also patients with Fuchs' heterochromic uveitis syndrome, which can follow infection with either Rubella virus or Cytomegalovirus, have high incidences of *Toxoplasma*-like lesions [55, 56]. Furthermore, we do not know how long immunoreactivity can persist after the clinically active phase of the disease has abated or how specific the observed local immunoreactivity is. And we are still ignorant of the role played by the mode or route of infection. Finally, we do not know whether reinfection with a different and more virulent strain is the trigger for inducing or reactivating the ocular disease [19, 57].

Whether the strain of *Toxoplasma gondii* has a bearing on the detection of local antibody production has not been determined. However, the highly virulent type I strains are typically associated with ocular toxoplasmosis in healthy Brazilian individuals [58], whereas the less virulent ones can induce ocular disease only in immunocompromised patients. However, the highly virulent type I strain may also occur in European and North American patients who are suffering from ocular toxoplasmosis, which complicates the situation [59]. It is conceivable that the less virulent type II strains are associated with patients who have contracted ocular toxoplasmosis congenitally However, available strain-typing data do not support this contention [60, 61]. Further studies are required to determine the relationship between *Toxoplasma* strains and the outcome of laboratory analyses.

## ALGORITHM FOR A CLINICALLY TAILORED LABORATORY ANALYSIS

**FIGURE 2:**
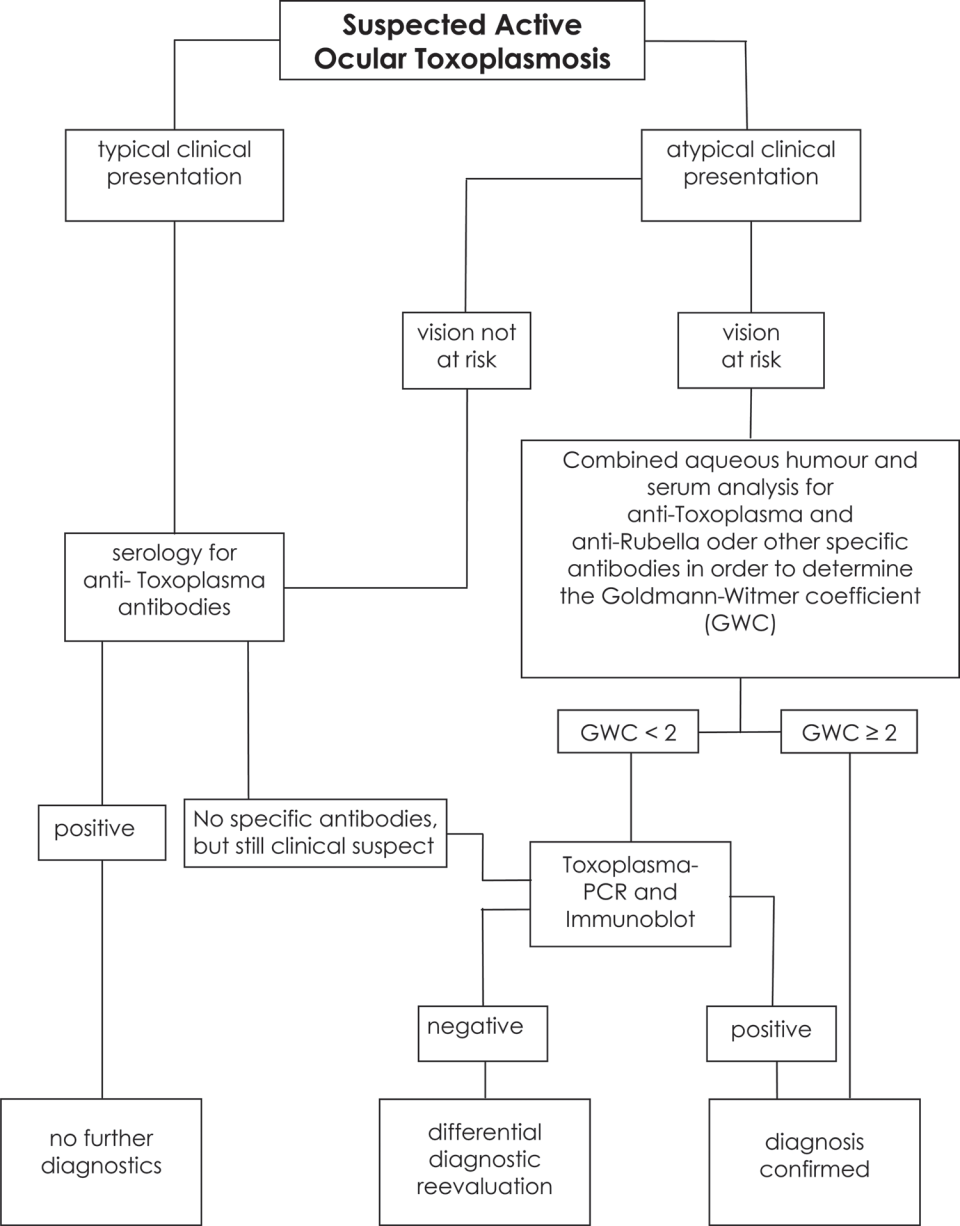
Algorithm to a clinically tailored laboratory analysis in suspected ocular toxoplasmosis.

On the basis of the available information, we have developed an algorithm for the laboratory confirmation of clinically suspected cases of ocular toxoplasmosis (Figure 2). If the retinal lesions are typical for toxoplasmic retinochoroiditis, if samples of serum register positive for *Toxoplasma*-specific IgG and negative for Toxoplflsma-specific IgM, and if the patient responds to appropriate anti-*Toxoplasma* therapy, most authorities will agree that the individual is suffering from a reactivated form of ocular toxoplasmosis. If serum samples are positive for *Toxoplasma*-specific IgM, additional laboratory testing is recommended to establish whether ocular involvement is the consequence of a recently acquired infection. Sampling of the intraocular fluids is not required in either instance. However, if there is clinical doubt about the diagnosis, paired samples of aqueous humor and serum should be collected and analyzed in parallel.

### Positive

Investigation of these samples may face technical difficulties since none of the commercially available ELISA, PCR, and immunoblotting tests are standardized for testing ocular fluid samples, nor are they routinely applied in most commercial laboratories. Since the number of cases per center requiring the aforementioned diagnostics are few, a cooperation with one of the specialized diagnostic centers should be considered. In these centers a support in the clinical interpretation of outcomes may also be available.

If local IgG production is detected using the ELISA technique (Goldmann-Witmer coefficient), then the clinical diagnosis can be considered confirmed. If no local specific IgG production is detected, or if the blood–retinal barrier is severely compromised, we recommend an immunoblotting analysis of the serum and aqueous humor (as a more sensitive alternative), and a PCR analysis of the latter to detect parasitic DNA. Using this strategy, a laboratory confirmation of the diagnosis can be achieved in 85% of cases (Figure 2). If a laboratory confirmation is required for the remaining cases, the aforementioned evaluations can be supplemented with similar analyses of the vitreous. If the patient has undergone no treatment, vitreal or chorioretinal biopsies can be subjected to cytological, immunocytological, PCR, and cell-culturing analyses [35].

## CONCLUSION

In conclusion, the clinical diagnosis of ocular toxoplasmosis may be supported by laboratory tests in 60–85% of cases, depending on the time of sampling. Analysis of the aqueous humor is particularly helpful in patients with atypical lesions or in individuals who are irresponsive to anti-*Toxoplasma* therapy. Even so, a laboratory confirmation of the clinical diagnosis is not achieved in 15–40% of cases.

Several aspects of humoral immunity are still poorly understood. These include (1) unclariries with regard to the diagnostic window of false-negative results; (2) the discordance in samples registering positive for antibodies that are detected by the immunoblotting and ELISA techniques; (3) the mechanism underlying the persistence of antibody production after the cessation of disease activity; and (4) the question as to whether an unspecific stimulator of the humoral immune response is capable of inducing the local production of specific antibodies. Although little is known of the role that is played by the parasitic strain in the evolution of the disease, sufficient evidence has accumulated to indicate that this factor has been hitherto underestimated. To further our understanding of the pathophysiology of ocular toxoplasmosis, these issues must be addressed and clarified.
