# Erratum

**DOI:** 10.3109/09273948.2011.633373

**Published:** 2011-11-22

We have missed an error in *Ocular Immunology & Inflammation*, 19(4), 255-261, 2011, DOI: 10.3109/09273948.2011.595872.

On p. 258 of the article entitled “Diagnostic Approach to Ocular Toxoplasmosis,” by J. G. Garweg et al., the heading “Positive” was accidentally introduced into the text. This heading is meaningless and should be disregarded.

